# Faith Practices Reduce Perinatal Anxiety and Depression in Muslim Women: A Mixed-Methods Scoping Review

**DOI:** 10.3389/fpsyt.2022.826769

**Published:** 2022-05-24

**Authors:** Shannon D. Simonovich, Nadia Quad, Zehra Kanji, Karen M. Tabb

**Affiliations:** ^1^School of Nursing, College of Science and Health, DePaul University, Chicago, IL, United States; ^2^Department of Biological Sciences, College of Science and Health, DePaul University, Chicago, IL, United States; ^3^School of Social Work, University of Illinois at Urbana-Champaign, Urbana, IL, United States

**Keywords:** pregnancy, postpartum, mental health, faith, Muslim

## Abstract

Higher rates of depression and anxiety are reported among women who belong to racial and ethnic minority groups, contributing to adverse birth outcomes, and remains a taboo topic within the global Muslim community. Non-pharmacological coping mechanisms such as prayer may be employed to reduce perinatal depression and anxiety, however the literature is sparse on the use of this intervention among pregnant Muslim women. Therefore, we aimed to conduct a scoping review examining the use of Muslim faith practices on anxiety and depression in perinatal period. Nine studies were identified that demonstrate that Muslim faith practices reduce perinatal anxiety and depression symptoms. These studies demonstrate that prayers and other faith-based practices, including reciting parts of the Quran, saying a Dua, and listening to audio recordings of prayers are all effective in decreasing anxiety, depression, stress, pain and fear in Muslim women during pregnancy, during childbirth, during an unexpected cesarean section, and when experiencing infant loss. Despite the scoping review's small sample size, findings confirm that incorporation of faith practices effectively reduces perinatal depression and anxiety among Muslim women and should be utilized in clinical settings for non-pharmacological management of perinatal mood disorders.

## Introduction

Perinatal mental health disorders are experienced by 10% of pregnant women across the globe (1). Rates of both depression and anxiety are higher among pregnant women who belong to racial and ethnic minority groups, with 17% experiencing depression, and 19% experiencing anxiety, respectively (2). It is well-documented that depression and anxiety during the perinatal period are associated with an increased risk of adverse birth outcomes such as preterm birth and low birth weight, particularly among women from racial minority groups (3, 4). Risks for poor obstetric and neonatal health outcomes in perinatal populations with mental health concerns are exacerbated by “concomitant conditions” including substance use, poverty and domestic violence (5). Despite the significant personal and societal cost of perinatal mood disorders, identification and treatment of perinatal anxiety and depression remain sparse with less than half of women diagnosed with major depression receiving treatment (6).

Muslim women are part of the fastest-growing religious group in the world, with 24%, 1.8 billion people, identifying as Muslim (7). The mental health of Muslim women during the perinatal period is particularly important to address given the pervasive stigma surrounding mental health in this community which creates barriers to treatment (8). Muslim women may also be at increased risk for stress and anxiety during pregnancy by “easily being identified as different from others due to the observance of the hijab” (9). Muslims experience “ongoing discrimination and self-stigma” similar to that seen in other racial, ethnic and religious communities and report lower rates of accessing mental health services (10–12). The social stigma surrounding perinatal mental health in the Muslim community, paired with inequitable access to care, may further health disparities experienced by this community.

Islamic faith practices are impactful in the daily lives of the Muslim community and vary among individuals but are always performed with intention for Allah, the Arabic word for God. Common Islamic faith practices include structured prayers and rituals, reciting the Qur'an, remembrance of Allah referred to as dhikr, and saying a Dua in which individuals request guidance, make a personal appeal or invocation “either on behalf of another or for oneself” (13). In Islam, prayers and faith-based practices are used to help overcome difficult times, including times of pain and fear, and become closer to a higher power (14). While perinatal mental health may remain a taboo topic in the Muslim community, the use of Islamic faith practices to reduce anxiety and depression has been demonstrated in the general population studies and among cancer patients (15, 16). There has also been recent study of the relationship between perceived stress, religious coping and religiosity among migrant Muslim women (9). However, the relationship between Islamic faith practices and perinatal anxiety and depression has not been described to date. Therefore, the purpose of this scoping review was to examine and summarize the efficacy and utility of faith practices on perinatal anxiety and depression in Muslim women during the antenatal and postnatal periods.

## Materials and Methods

### Study Design and Eligibility Criteria

This scoping review utilized Arksey and O'Malley's methodology to systematically review the literature pertinent to this topic (17). Eligibility criteria, databases, inclusion and exclusion criteria were developed with the full study team a priori. Measurement of utilization of Islamic faith practices and perinatal mood disorders including anxiety and depression directed our study design. The research question guiding our study was, “What effect do Islamic faith practices have on anxiety and depression in Muslim women during the perinatal period, including during pregnancy and the postpartum period?” For inclusivity in study design as the first scoping review to date on this emerging topic all published peer-review journal articles written in English were considered for inclusion in our scoping review. Measurement of Islamic faith practices was broad to capture all original research studies that focused on activities including both active and passive exposure to Qur'an recitation. Measurement of perinatal mood disorders included both formal assessment of anxiety or depression during pregnancy and postpartum via scale measures as well as self-report data.

**Figure 1:**
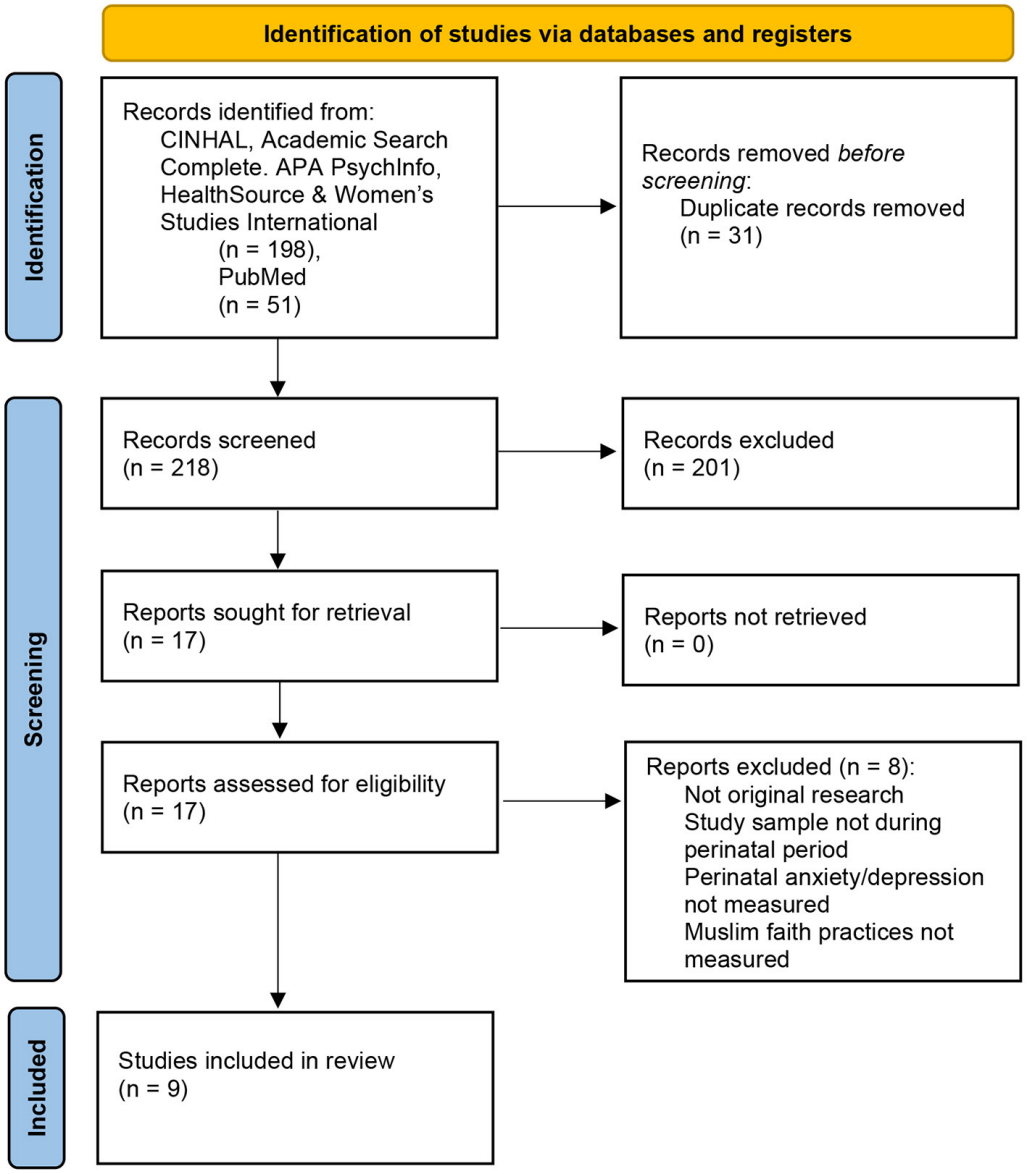
Scoping Review Flowchart PRISMA 2020 flow diagram for new systematic reviews which included searches of databases and registers only. For more information, visit: http://www.prisma-statement.org/ (1).

Databases utilized for this scoping review included PubMed, and each of the five EBSCOhost databases: CINAHL Complete, Academic Search Complete, APA PsycInfo, Healthsource: Nursing/Academic Edition, and Women's Studies International. Search strategy in PubMed was completed using a combination of the following keywords: (mental health OR mental illness OR mentally ill OR depression OR depressive OR depressed OR anxiety) AND (pregnant OR pregnancy OR perinatal OR antepartum OR prepartum OR antenatal OR antenatally OR prenatal OR prenatally) AND [Muslim(my) OR Muslim(tiab) OR Islam(mh) OR islam (tiab) OR Islamic (tiab) OR Sunni (tiab) OR Shia (tiab) OR Quran (tiab)]. Search strategy in all other databases utilized a combination of the following keywords: (mental health OR mental illness OR mentally ill OR depression OR depressive OR depressed OR anxiety) AND (pregnant OR pregnancy OR perinatal OR antepartum OR prepartum OR antenatal^*^ OR prenatal^*^) AND [TI (Muslim OR islam^*^ OR Sunni OR Shia OR Quran) OR AB (Muslim OR Islam^*^ OR Sunni OR Shia OR Quran) OR SU (Muslim OR islam^*^ OR Sunni OR Shia OR Quran)]. Database searches were completed from July 2020 to September 2020. All peer-reviewed articles with publication dates through September 2020 were included for examination. The data collection and screening process began with a total of 249 abstracts and resulted in a total of 9 included peer-reviewed articles (Figure 1).

### Literature Screening

Literature screening began with review of the abstracts, utilizing RAYYAN. Each abstract was reviewed independently by two content experts (NQ, ZKG) for the following inclusion criteria: (1) measurement of Islamic faith practices, (2) measurement of mood disorders, specifically anxiety and/or depression, including use of descriptive behaviors such as “anxious thoughts, worry, rumination, nerves” (3) population of pregnant women up until delivery, (4) original research study in peer-reviewed publication, (5) available in English. Discordant results for abstracts as well as “maybe” articles were reviewed in tandem by two perinatal mental health research scientists (SDS, KMT). For full text review, articles were obtained for all those which met the inclusion criteria. Full text reviews were conducted by SDS and KMT with no disagreements.

## Results

Following completion of the scoping review, nine articles were included for synthesis on this research topic. In sum, 6 articles were quantitative in methodology while three were qualitative. The results of this scoping review are organized by methodological approach, first broadly describing quantitative findings followed by qualitative. Among the quantitative studies included in this scoping review, results are discussed in sum based upon the Muslim faith practice utilized as the intervention in the study design.

### Quantitative Studies Describing Islamic Faith Practices and Perinatal Mood

Across the six quantitative studies examined in this scoping review examining the relationship between perinatal anxiety and depression and Muslim faith practices as non-pharmacological interventions, four studies were completed in Iran, with the remaining 2 studies conducted in Indonesia from 2016 to 2020 (Table 1). All quantitative studies, 5 intervention-based and 1 cross-sectional design, were conducted during pregnancy.

**Table 1:** Characteristics of quantitative studies.

**Author(s), year**	**Study location**	**Study design**	**Sample size**	**Perinatal mood outcome examined**	**Type of Muslim faith practice**	**Timing of intervention**	**Intervention frequency**	**Formal measures**	**Outcomes and effect size**
Pakzad et al. (18)	Iran	Cross-sectional	300	Depression, anxiety, stress	Islamic Lifestyle Questionnaire	N/A	No intervention	Islamic lifestyle questionnaire and DASS-21	Significant inverse relationship between DASS-21 mental health scores and Islamic lifestyle questionnaire (r = −0.31); mean score of mental health measure decreases 0.09 per unit increase in Islamic lifestyle score.
Hamidiyanti and Pratiw (19)	Indonesia	Non-blind RCT	30	Anxiety	Listening to Qur'an recitation recording	28–34 weeks	15 min, three times per week for 4 consecutive weeks	Hamilton Anxiety Rating Scale (HARS)	Anxiety scores significantly reduced post-intervention. HARS mean post-intervention 12.88 (1.31) in intervention group vs. 15.06 (0.77) in control group (p <0.01)
Irmawati et al. (20)	Indonesia	Quasi-experimental, pre-test post-test	40	Anxiety	Listening to Qur'an recitation recording	During first stage of labor	one time intervention, during labor	anxiety measure, also level of cortisol and time of labor	Friedman-test demonstrates significant reduction in level of anxiety (*p* < 0.001). Level of cortisol and time of labor were also significantly lower (*p* < 0.001).
Jabbari et al. (21)	Iran	RCT	168	Stress, anxiety, depression	Listening to Qur'an recitation recording	Second trimester	20 min sessions over 3 weeks	EPDS, STAI, PSS	After intervention, scores of perceived stress, anxiety and depression were significantly lower in both translation group and non-translation Qur'an group compared to control.
Aslami et al. (22)	Iran	Quasi-experimental, pre-test post-test	75	anxiety, depression	mindfulness protocol based on Islamic-Spiritual Schemes vs. cognitive behavioral therapy	16–32 weeks	8 weeks, maximum 2-hour session per week	Beck anxiety and depression questionnaire	Difference between average mindfulness group and CBT group in anxiety was −12.133 and in depression variable was −10.53 (*p* = 0.001).
Mokhtaryan et al. (23)	Iran	RCT	84	Anxiety	Religious teaching on anxiety	20–28 weeks	6 weekly 60–90 min sessions	Spielberger anxiety scale, RAS-R	Significant different between intervention and control groups both after the intervention and 2 months after the study (*P* ≤ 0.001).

Among the five intervention studies, including randomized control trials and quasi-experimental designs, various interventions were utilized including listening to Qur'an recitation recordings in three studies (19–21), and educational interventions around mindfulness, anxiety and Muslim faith in two studies (22, 23). Timing of interventions included 4 studies during the second and early third trimesters, ranging from 16 weeks gestation to 34 weeks gestation, and one study during the first state of labor (20).

There were various measures of anxiety, depression and stress present among these 6 studies including DASS-21, the Hamilton Anxiety Rating Scale, EPDS, STAI, PSS, the Beck anxiety and depression questionnaire, the Spielberger anxiety scale, and RAS-R. There were no two studies that utilized the same measure of mental health in pregnant women. Among the 5 intervention studies, all utilized pre-test post-test design with two or more measures for mental health throughout pregnancy. Across these 5 peer-reviewed intervention studies, results consistently reported significant reductions in mental health scores from the pre-intervention to post-intervention time points. Hamidiyanti and Pratiwi (19) intervention of listening to Qur'an recitation recordings from 28 to 34 weeks, 3 times a week for 4 weeks found that HARS anxiety scores significantly reduced post-intervention with HARS mean post-intervention 12.88 (1.31) in intervention group vs. 15.06 (0.77) in control group (*p* < 0.01). Jabbari et al.'s (21) intervention study of Qur'an recitation recordings during the second trimester, in 20-min sessions over 3 weeks, scores of perceived stress, anxiety and depression were significantly lower in comparison to the control group. In Irmawati et al. (20) intervention study of listening to Qur'an recitation recordings during the first state of labor, Friedman-test demonstrates significant reduction in level of anxiety (*p* < 0.001) with significant reduction in cortisol levels and time of labor as well (*p* < 0.001).

The two educational interventions around mindfulness, anxiety and Muslim faith reported similarly significant results. Aslami et al.'s (22) study of the impact of a “mindfulness protocol based on Islamic-Spiritual Schemes” in comparison to traditional cognitive behavioral therapy exposed pregnant women to 8 weeks of 2-h per week session from 16 to 32 weeks gestation, utilizing the Beck anxiety and depression questionnaire to assess perinatal mood. Study findings demonstrated statistically significant difference between average mindfulness group and CBT group in anxiety was −12.133 and in depression variable was −10.53 (*p* = 0.001). Mokhtaryan et al.'s (23) study of perinatal anxiety focused on religious teaching on anxiety based upon Islamic principles, with pregnant women from 20 to 28 weeks including 6 weekly sessions lasting 60–90 min each, utilizing the Spielberger anxiety scale and RAS-R as formal measures of mental health. Pre-intervention to post-intervention found statistically significant reductions in anxiety both directly after the intervention as well as 2 months after the study (*P* ≤ 0.001).

One study included was cross-sectional in nature, with a sample size of 300, in which pregnant women completed the Islamic Lifestyle Questionnaire and DASS-21. Correlation analyses noted that a statistically significant inverse relationship between mental health scores and responses to the Islamic lifestyle questionnaire (r = −0.31) with regression analyses demonstrating that a “mean score of mental health measure decreases 0.09 per unit increase in Islamic lifestyle score” (18). This study finding indicates that there may be an overall correlation between lower mental health scores in Muslim women who self-report a higher rate of Islamic faith practices.

### Qualitative Studies Describing Islamic Faith Practices and Perinatal Mood

Qualitative results of the scoping review include three original research articles examining the relationship between Muslim faith practices and perinatal mood during the postpartum period (Table 2). Mutmainnah et al.'s Indonesian study published in 2019 reported that “doing dhikr and reciting Al Qur'an” were utilized as effective coping mechanisms to reduce perinatal anxiety in pregnant Muslim women, promoting how women felt “calm and peaceful doing so.” The remaining two qualitative studies examining Muslim faith practices and perinatal mood were completed with postpartum women who had experienced loss of their child (25, 26). While the two studies were completed in Somaliland and Malaysia, respectively, both studies consistently reported that Muslim faith practices including recitation of the Qur'an and reading Dua reduced perinatal anxiety, fear and worries supporting the women in processing their grief and easing sorrow around loss, noting that “they overcame the hardship by praying to Allah” (25) and “felt calm” during Muslim prayer recitation (26).

**Table 2:** Characteristics of qualitative studies.

**Author(s), year**	**Study location**	**Study design**	**Sample size**	**Sample characteristics**	**Outcomes**
Mutmainnah and Afiyanti (24)	Indonesia	One-on-one interview, single session	7	Postpartum women, youngest child 2 months to 1 year, age 24–31, self-identified as Muslim	5 of 7 participants reported that doing dhikr and reciting Al Qur'an as a coping mechanism for reducing perinatal anxiety.
Osman et al. (25)	Somaliland	One-on-one interview, single session	10	Postpartum women, within 6 months of birth experienced stillbirth at or after 28 weeks gestation, age 17–43, 5 primipara, 5 multipara	Participants reported Muslim prayer reduced anxiety, fear and worries around stillbirth and child loss.
Sutan and Miskam (26)	Malaysia	Multimethod: One sample completed series of one-on-one interviews, one sample completed a single focus group interview, a third sample completed an in-depth interview within a week following a perinatal loss	16 individual women, 5 women in focus group, 10 sets of parents	Postpartum women, within 6 to 12 months following perinatal loss, as well as couples within a week following a loss.	Religious practices of reciting the Qur'an and reading Dua described as notable aspect of support during grief to ease sorrow of perinatal loss.

## Discussion

This scoping review included nine quantitative and qualitative studies which consistently described the utility of faith practices in reducing perinatal anxiety and depression symptoms in a global sample of Muslim women. Specifically, the studies in this review, found overwhelmingly that prayers and other faith-based practices, including but not limited to reciting parts of the Quran, saying a Dua, or just the simple act of listening to prayers on a smart phone, aided in decreasing stress, pain, and fear in Muslim women before and during childbirth, when experiencing perinatal loss, and during an unexpected cesarean section surgery (19, 23).

Prayer, as a healing practice for mood disorders, has been well-documented in many religious groups (Kirk) (27). Literature to date reports the utility of faith-based coping mechanisms in perinatal populations with Lara-Cinisomo and colleagues' work describing religiosity as protective postpartum against depression in Latina women (28). Furthermore, Keefe and colleagues' qualitative examination of the relationship between faith and spirituality among Black and Latina mothers reinforces that faith practices can be a source of peace for postpartum women who have a history of postpartum depression (29). While non-pharmacological coping mechanisms such as prayer have been demonstrated to reduce perinatal depression and anxiety in Christian childbearing individuals, this scoping review is the first known mixed methods publication of the utility and efficacy of faith practices in reducing perinatal mood symptoms among Muslim women.

Research to date highlights the importance of investment in public health interventions paired with additional perinatal mental health services, to reduce mental health related maternal child morbidity and mortality (5). Non-pharmacological interventions that rely upon individuals' existing values and coping mechanisms, such as faith practices, may be one free and effective public health intervention to reduce perinatal anxiety and depression across numerous religious groups. With perinatal mental health disorders rarely being discussed among the Muslim community and other religious groups, healthcare providers can educate patients and their families about the use of faith practices to reduce perinatal anxiety and depression symptoms at the time of screening. This non-pharmacological intervention should be made available to both antenatal and postpartum populations when faced with periods of stress or difficulty, particularly among women uninterested in pharmacological interventions for mental health symptoms.

Increased cultural awareness around faith practices for diverse populations should be included in training for patient-facing members of healthcare organizations and public health systems including obstetricians and gynecologists, certified nurse midwives, nurses, social workers, public health providers and patient care support staff. Future research should consider testing the utility of audio or visual recordings of prayer, from different global faith traditions, in the reduction of perinatal anxiety and depression symptoms during in-patient hospital stays. Integration of faith practice recordings on hospital televisions would allow bedside clinicians and support staff to make this intervention readily available to those interested and enhance the utilization of this coping mechanism when preparing for birth or experiencing perinatal loss. Discussion of faith practices as public health screenings may increase a sense of community support around this issue, as faith-based multimedia is available for download directly onto individuals' personal mobile devices.

### Limitations

Despite the number of strengths in this review, a potential limitation of this scoping review includes the limited number of studies to date, that discuss the correlation between perinatal mood disorders and faith-based practices used by Muslim women. Additionally, most studies included in this review paper take place in primarily Muslim countries. More research is needed to understand perinatal mood disorders in Muslim women who have moved and assimilated in new areas.

## Conclusion

Consideration of this study's findings should also include creating in-patient hospital environments that are respectful of women and their families during prayer. Supporting the utilization of faith practices in Muslim women, and others, during the intrapartum period may include thoughtfully allowing privacy during prayer, temporary removal of continuous monitoring devices, and asking if patients would like to request religious service support from the hospital directory of faith leaders. Acknowledging to antenatal and postpartum individuals that their faith practices are available to them as a resource and may be helpful in relieving perinatal mood symptoms may create an opportunity building trust and rapport with pregnant patients and their families.

## Author Contributions

SDS, NQ, and KT conceived and designed the study. NQ and ZK performed the search strategy and abstract review process, with supervision by SDS and KT. SDS, NQ, ZK, and KT wrote the first draft and contributed to the final draft. SDS and NQ wrote the introduction and wrote the methods. SDS wrote the results. SDS, ZK, and KT wrote the discussion. All authors viewed and approved the final version.

## Conflict of Interest

The authors declare that the research was conducted in the absence of any commercial or financial relationships that could be construed as a potential conflict of interest.

## Publisher's Note

All claims expressed in this article are solely those of the authors and do not necessarily represent those of their affiliated organizations, or those of the publisher, the editors and the reviewers. Any product that may be evaluated in this article, or claim that may be made by its manufacturer, is not guaranteed or endorsed by the publisher.
